# Knockdown of HDAC1 expression suppresses invasion and induces apoptosis in glioma cells

**DOI:** 10.18632/oncotarget.18227

**Published:** 2017-05-26

**Authors:** Xiao-Qiang Wang, Hong-Min Bai, Shi-Ting Li, Hui Sun, Ling-Zhao Min, Bang-Bao Tao, Jun Zhong, Bin Li

**Affiliations:** ^1^ Department of Neurosurgery, Xinhua Hospital Affiliated to Shanghai Jiao Tong University School of Medicine, Shanghai 200092, China; ^2^ Department of Neurosurgery, Guangzhou General Hospital of Guangzhou Military Command, Guangzhou 510010, China

**Keywords:** glioma, HDAC1, proliferation, invasion, prognosis

## Abstract

Glioma is the most common malignant tumor of the central nervous system, with a low survival rate of five years worldwide. Although high expression and prognostic value of histone deacetylase 1 (HDAC1) have been recently reported in various types of human tumors, the molecular mechanism underlying the biological function of HDAC1 in glioma is still unclear. We found that HDAC1 was elevated in glioma tissues and cell lines. HDAC1 expression was closely related with pathological grade and overall survival of patients with gliomas. Downregulation of *HDAC1* inhibited cell proliferation, prevented invasion of glioma cell lines, and induced cell apoptosis. The expression of apoptosis and metastasis related molecules were detected by RT-PCR and Western blot, respectively, in U251 and T98G cells with *HDAC1* knockdown. We found that HDAC1 knockdown upregulated expression of BIM, BAX, cleaved CASPASE3 and E-CADHERIN, and decreased expression of TWIST1, SNAIL and MMP9 in U251 and T98G cells with *HDAC1* knockdown. *In vivo* data showed that knockdown of *HDAC1* inhibited tumor growth in nude mice. In summary, HDAC1 may therefore be considered an unfavorable progression indicator for glioma patients, and may also serve as a potential therapeutic target.

## INTRODUCTION

Malignant glioma, the most common and leading cause of death in cancer regarding the central nervous system in adults, accounting for 45 to 55% of primary intracranial tumors and has been classified according to the 2007 World Health Organization (WHO) classification system as grades II-IV [1]. Glioblastoma multiforme (GBM, WHO grade IV) is the most common and biologically aggressive malignant glioma. However, the overall prognosis of patients with GBM remains poor, with the median survival rate less than 12 months after diagnosis because they are resistant to chemotherapeutic agents [2]. Despite surgical resection and radiotherapy are crucial to the success of treatment, the metastasis of malignant glioma cells represents one of the major obstacles for therapy. The invasiveness characterization of glioma is produced by uncontrolled cellular proliferation, rapid diffuse infiltration, and high apoptosis resistance of gliomas [3, 4]; however, little is known about the factors that mediate glioma invasion. Thus, understanding the molecular mechanism involved in glioma tumorigenesis and progression is important for developing novel therapeutic strategies for glioma.

Histone deacetylation is maintained by histone deacetylases (HDACs), which synergistic inhibition of the activity of gene transcription with DNA methyl transferases (DNMTs) responsible for DNA methylation [5]. HDACs in humans are grouped into four classes based on their homology to yeast HDACs, including structure and cellular localization. Classes I, II and IV comprise Zn^2+^ dependent enzymes, and Class III comprises Zn^2+^ independent, NAD-dependent enzymes [6]. Numerous correlational studies reported that the HDACs expression was abnormal in human tumors [7], among which HDAC1, HDAC5, and HDAC7 can be used as molecular markers in early diagnosis of cancer, treatment targets and judgment of prognosis [8]. HDACs overexpression was associated with a reduction in both disease-free survival and overall survival and was shown to predict prognostic value in patients [9, 10]. HDAC1 belongs to HDAC class I and is elevated in a variety of cancers, such as gastric [11], colorectal [12], lung cancer [13], bladder cancer [14]. Downregulation of HDAC1 induces cell cycle arrest, decreases viability, and increases apoptosis in cancer cells and fibroblasts [15–17]. Combined genetic knockout of HDAC1 and HDAC2 dose-dependently augmented spontaneous tumorigenesis via regulation of c-Myc collaborating genes and p53 function [18]. HDAC8 may be linked to PLCγ1, a signal transducer, absent activation and subsequent failure to release intracellular Ca^2+^ and reactive oxygen species (ROS) in cutaneous T-cell lymphoma cells, suggesting that HDAC8 may play a role in the induction of apoptosis in T cell lymphomas [19].

Significant nuclear expression of HDAC1 has been found in glioma cells during tumor recurrence and malignant tumor progression [20]. Previous studies have reported that high HDAC1 expression was correlated with an invasive and proliferative phenotype of GBM cells [21]. Moreover, HDAC1 inhibitor for the treatment of different types of cancer, including GBM, not only as a single agent, but also in combination with other anticancer agents, has been widely tested in clinical trials [22–25]. However, the relationships between HDAC1 and apoptosis, as well as between HDAC1 and invasion of malignant glioma tumors, are not available.

In the present study, we demonstrated that overexpression of HDAC1 in human glioma tissues and cell lines was associated with advanced WHO grade, low index of MIB (%) and poor prognosis of glioma patients. Knockdown of *HDAC1* can inhibit cell proliferation, inhibit invasion of glioma cell lines, and induce cell apoptosis. Moreover, gene set enrichment analysis (GSEA) using The Cancer Genome Atlas (TCGA) dataset showed that HDAC1 was positively related to apoptosis and metastasis pathways, which was further validated in glioma cell lines with *HDAC1* knockdown. Finally, *HDAC1* knockdown inhibited tumor growth in nude mice *in vivo*. Our data provide significant molecular insight into HDAC1 and its regulation mechanisms in gliomas.

## RESULTS

### HDAC1 overexpression in human glioma tissues

**Figure 1:**
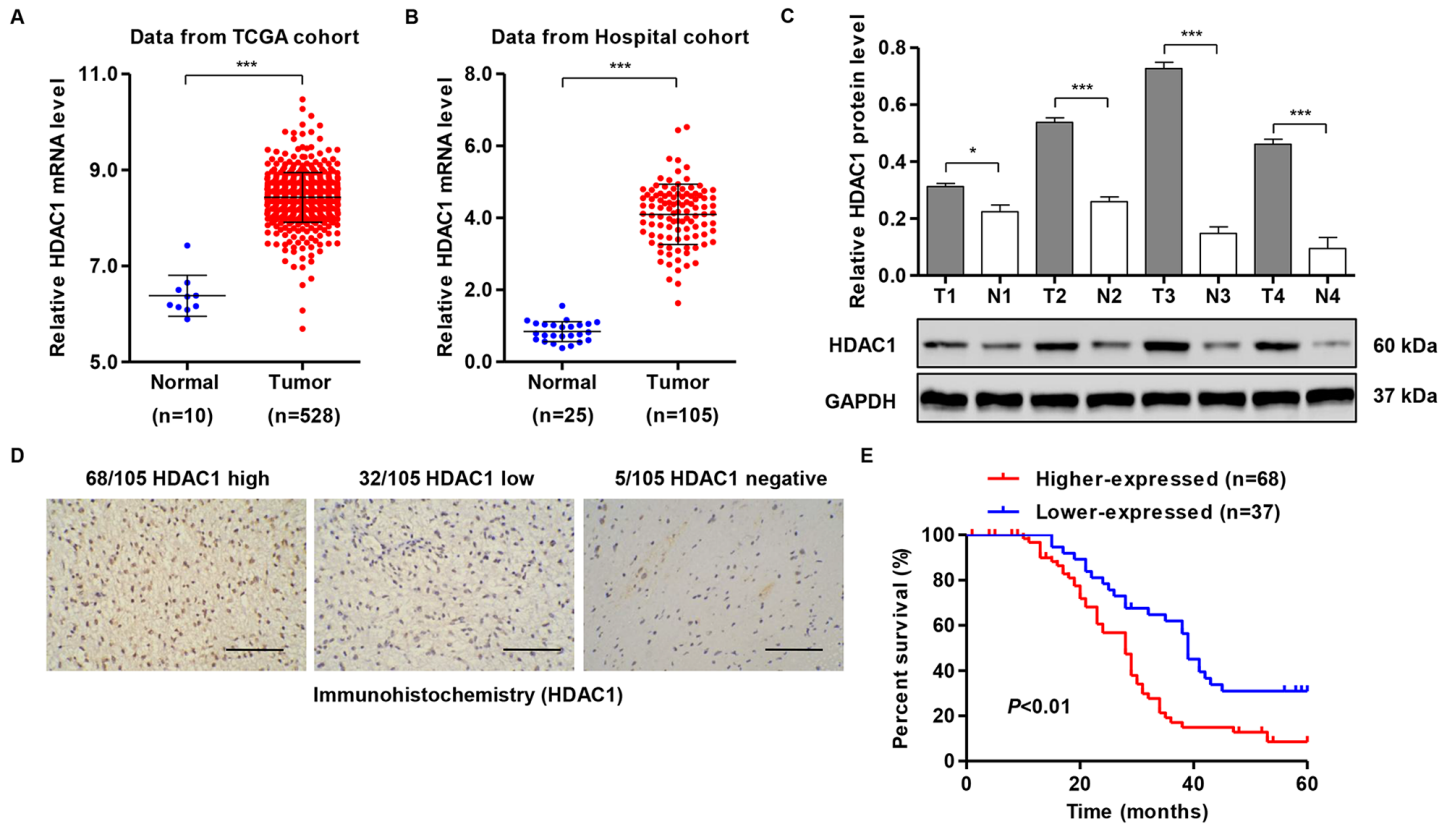
HDAC1 expression of patients with glioma **(A)** *HDAC1* mRNA levels were significantly higher in glioma tissues (n = 528) than in normal brain tissues (n=10) from the TCGA GBM dataset. **(B,C)** *HDAC1* mRNA and protein levels were significantly increased in glioma tissues (n = 105) compared with normal brain tissues (n=25) from the Xinhua Hospital. Representative Western blots (lower panel) and quantitative results (upper panel) are shown. **(D)** Expression of HDAC1 was determined by immunohistochemistry staining in glioma tissues. Scale bars: 100 μm. **(E)** The overall survival time of 105 patients with glioma. T: tumor tissue; N: normal brain tissue. *P < 0.05, ***P < 0.001 by the unpaired, two-tailed Student's t-test.

We detected the mRNA levels of *HDAC1* using high-throughput RNA-sequencing data from the GBM cohort of TCGA and observed increased *HDAC1* expression in glioma tissues compared with normal brain tissues (Figure 1A). Then, we analyzed the expression levels of *HDAC1* in 105 snap-frozen glioma tissues and 25 normal brain tissues using RT-PCR and Western blot assays. As shown in Figure 1B and 1C, HDAC1 was obviously increased in glioma tissues compared with normal brain tissues, at both mRNA and protein levels. To assess the protein levels of HDAC1 in glioma tissues, immunohistochemistry staining of HDAC1 was performed in 105 human glioma specimens. High expression, low expression and negative expression of HDAC1 were observed in 68, 32 and 5 cases of glioma, respectively (Figure 1D).

According to immunohistochemistry staining results, all 105 glioma tissue samples were divided into two groups: higher HDAC1 expression and lower HDAC1 expression. Then, the correlations of HDAC1 expression and special clinicopathological parameters and prognosis of glioma were analyzed, as shown in Table 1. Chi-squared tests showed that higher HDAC1 expression was obviously associated with the advanced WHO grade and low index of MIB (%). According to the log-rank test and Kaplan-Meier analysis, higher HDAC1 expression associated with a poor prognosis of patients with glioma (Figure 1E). However, we did not find notable associations between HDAC1 expression and patients’ age, gender and tumor size (Table 1).

**Table 1:** Clinicopathological characteristics and follow-up data of 105 patients with glioma

Characteristics	Number of patients/number analyzed (%)	HDAC1		*P*-value
		High (n=68)	Low (n=37)	
**Age (years)**				0.472
≥45	52/105 (49.5%)	31	21	
<45	53/105 (50.5%)	27	25	
**Gender**				0.613
Female	57/105 (54.3%)	32	25	
Male	48/105 (45.7%)	26	22	
**Tumor size (cm)**				0.416
≥4.5	61/105 (58.1%)	33	28	
<4.5	44/105 (41.9%)	25	19	
**WHO grade**				0.0032**
I/II	39/105 (37.1%)	12	27	
III/IV	66/105 (62.8%)	43	23	
**MIB (%)**				0.021*
≥5	79/105 (75.2%)	49	30	
<5	26/105 (24.8%)	8	18	

WHO: World Health Organization. *P<0.05, **P<0.01, Chi-square test.

### HDAC1 overexpression in human glioma cell lines

**Figure 2:**
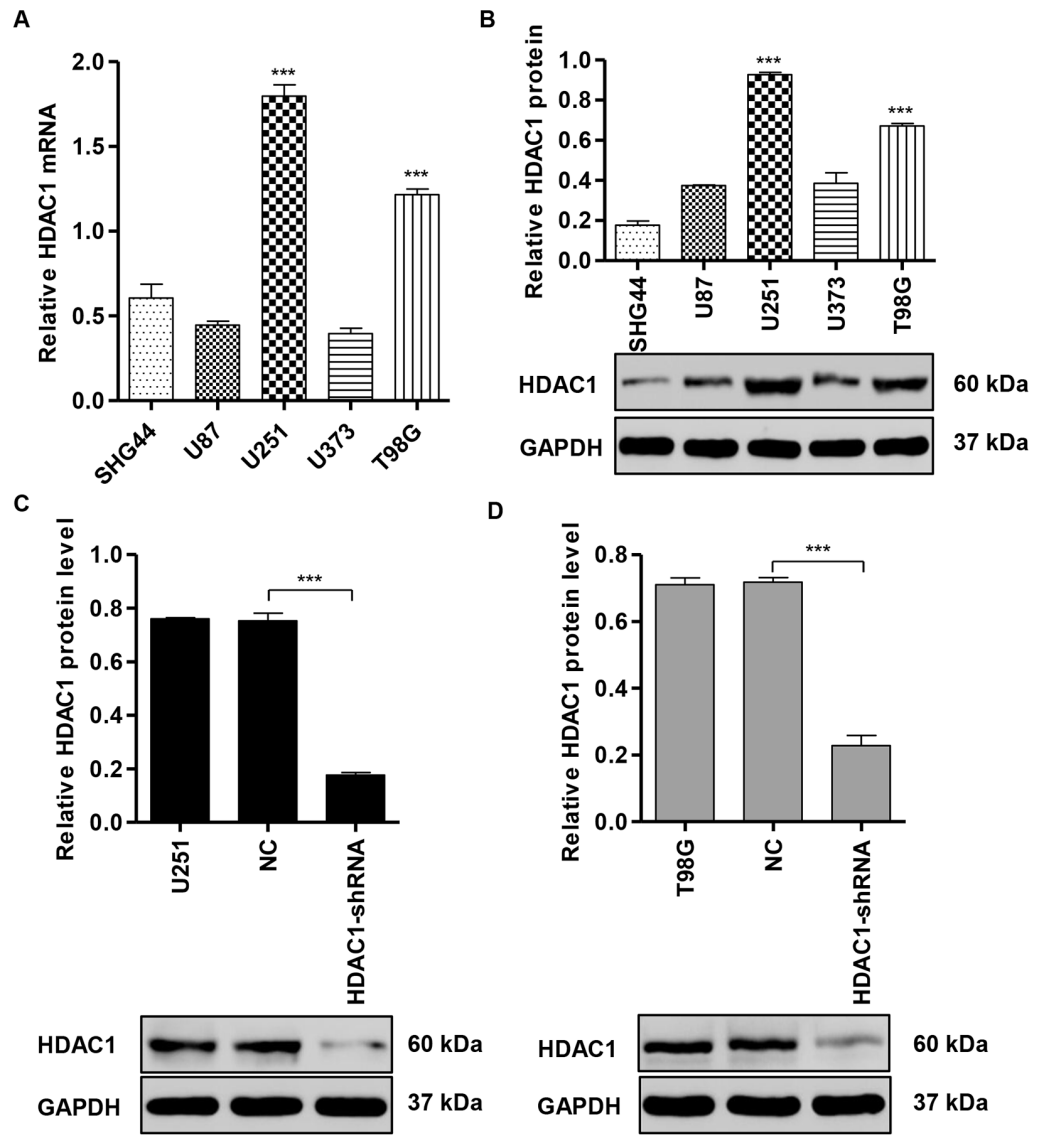
HDAC1 expression in glioma cell lines **(A,B)** *HDAC1* expression levels in five glioblastoma cell lines were analyzed by RT-PCR and Western blot. *GAPDH* was also detected as the internal control. Representative Western blots (upper panel) and quantitative results (lower panel) are shown. Knockdown of *HDAC1* by shRNA showed notably inhibited protein expression levels in **(C)** U251 and **(D)** T98G cells. Representative Western blots (upper panel) and quantitative results (lower panel) are shown. NC: pLVTHM-shRNA negative control infected cells. ***P < 0.001 by one-way ANOVA (**A, B**) and the unpaired, two-tailed Student's t-test (**C, D**).

To investigate the role of HDAC1 in glioma cell lines, we measured the expression of *HDAC1* in five glioblastoma cell lines using RT-PCR and Western blot assay. We found that*HDAC1* was significantly increased in U251 and T98G cells compared with another three glioblastoma cell lines at both mRNA (Figure 2A) and protein levels (Figure 2B). As a result of high expression of HDAC1 was associated with poor prognosis of patients with glioma, we suspected that HDAC1 might act as a potent oncogene in glioma. We therefore downregulated the expression of *HDAC1* in U251 and T98G cells by infection with pLVTHM-shRNA negative control (NC) or pLVTHM-HDAC1-shRNA in U251 and T98G cells. As shown in Figure 2C and 2D, pLVTHM-HDAC1-shRNA was able to efficiently suppress HDAC1 expression by 76.6% and 68.2% in U251 and T98G cells, respectively, whereas pLVTHM-shRNA negative control (NC) transfection in U251 and T98G cells had no effect on the HDAC1 expression.

### Knockdown of HDAC1 inhibits cell proliferation and induces apoptosis

**Figure 3:**
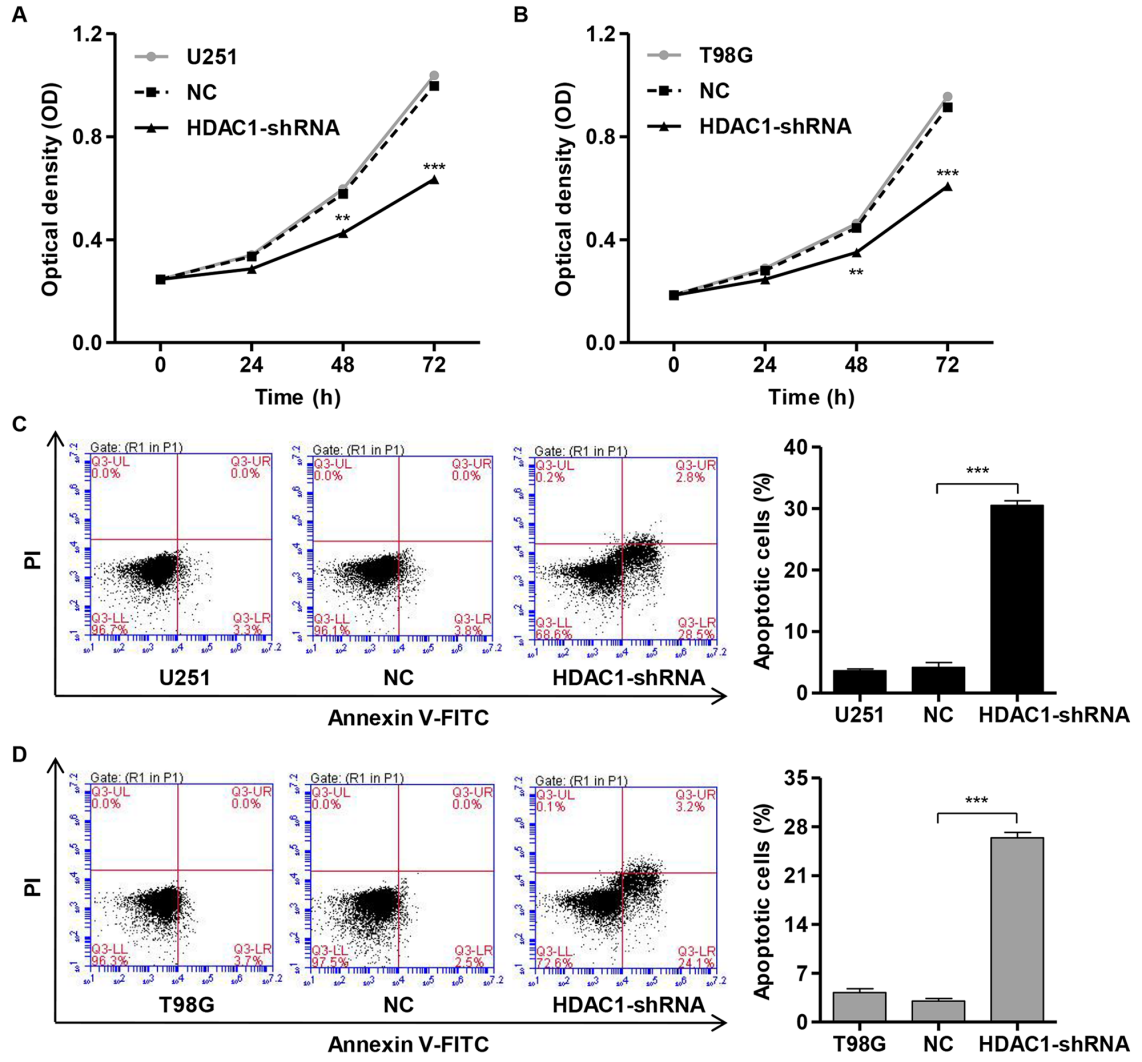
Knockdown of HDAC1 inhibits cell proliferation and induces apoptosis of glioma cell lines Cell proliferation was detected by CCK-8 assay in NC or pLVTHM-HDAC1-shRNA infected **(A)** U251 and **(B)** T98G cells. U251 and T98G cells were infected with NC or pLVTHM-HDAC1-shRNA and collected 48 h later. **(C)** U251 and **(D)** T98G cell apoptosis was analyzed by Annexin V/PI staining. NC: pLVTHM-shRNA negative control infected cells. **P < 0.01, ***P < 0.001 by the unpaired, two-tailed Student's t-test.

To investigate the role of *HDAC1* knockdown on the growth of glioblastoma cell lines, we performed CCK-8 assay to examine the proliferation of U251 and T98G cells. pLVTHM-HDAC1-shRNA infection significantly decreased the cell proliferation of U251 cells by 26.3% and 36.3% at 48 and 72 h and of T98G cells by 21.3% and 33.5% at 48 and 72 h, respectively (Figure 3A and 3B). Moreover, we also performed the Annexin V-FITC/PI staining and flow cytometry assay to assess the function of HDAC1 in apoptosis in glioblastoma cell lines by. Our findings showed that *HDAC1* down-regulation in U251 and T98G cells markedly increased cell apoptosis by approximately 7.2-fold and 9.9-fold, respectively, in comparision with corresponding NC cells (Figure 3C and 3D). Taken together, these data suggest an anti-proliferative and pro-apoptotic role of HDAC1-shRNA in glioblastoma cells.

### Knockdown of HDAC1 inhibits cell migration, invasion and adhesion

**Figure 4:**
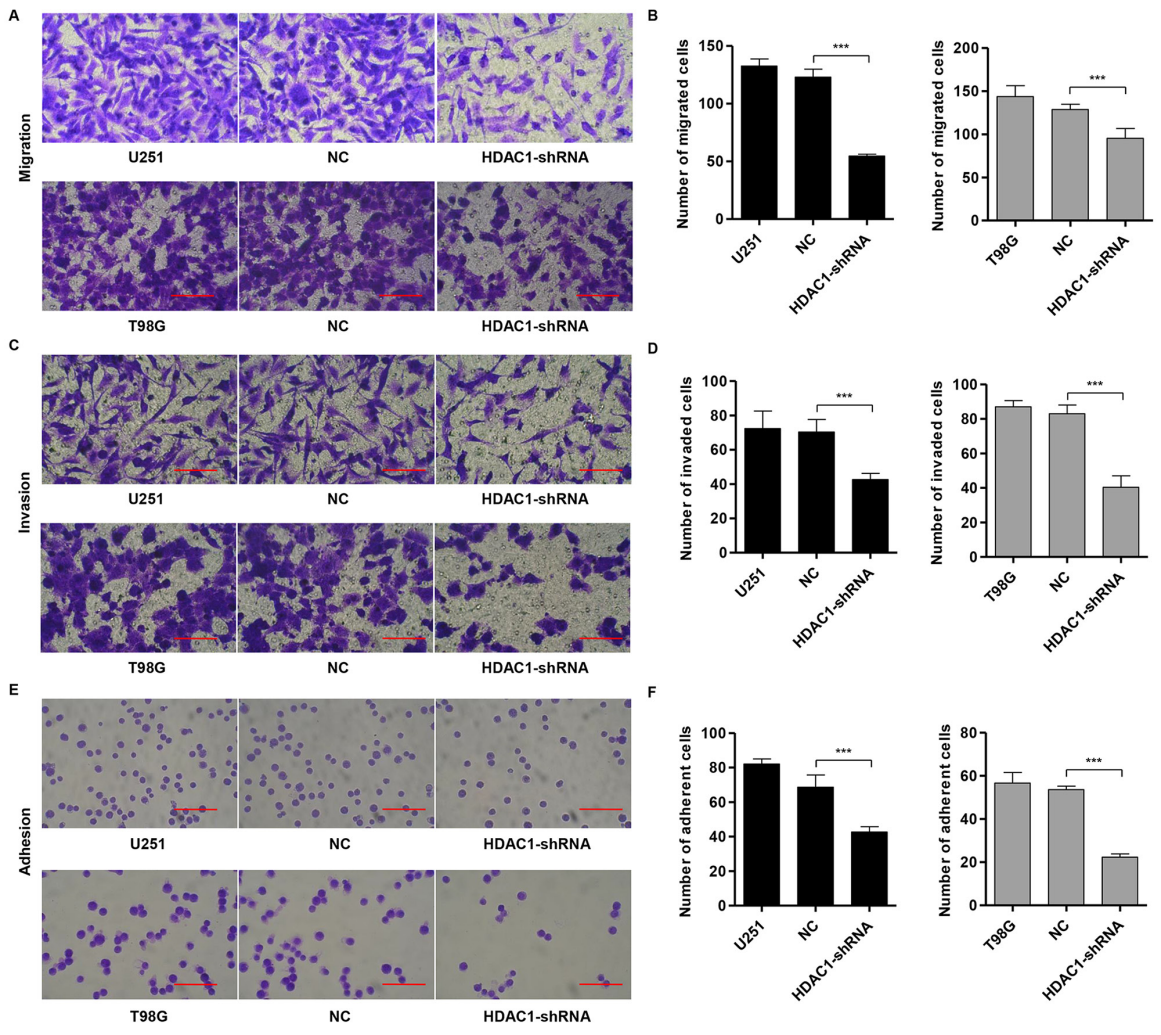
Knockdown of HDAC1 inhibits migration, invasion and adhesion in glioma cell lines U251 and T98G cells were infected with NC or pLVTHM-HDAC1-shRNA. **(A,B)** Cell migration, **(C,D)** invasion and **(E,F)** adhesion assay were performed. Representative images (left panel) and the quantification (right panel) are shown. NC: pLVTHM-shRNA negative control infected cells. Scale bars: 100 μm. ***P < 0.001 by the unpaired, two-tailed Student's t-test.

It has been reported that cell-cell (intercellular) and/or cell-matrix adhesion are tightly related to tumor invasion. To examine the role of HDAC1 in migration, HDAC1-shRNA, control and NC cells were cultured in a Boyden chamber. After 48 h of incubation, both HDAC1-shRNA-U251 (Figure 4A) and HDAC1-shRNA-T98G GBM cells (Figure 4B) showed significantly decreased migratory ability (54 ± 2 cells and 95 ± 11 cells, respectively) compared with the NC cells (123 ± 7 cells and 128 ± 6 cells).

Using a matrigel-coated transwell chamber, we measured the effect of HDAC1 on the changes in cell invasion. After 48 h of incubation, similar numbers of NC-infected cells invaded through the matrigel (U251: NC, 70 ± 7 cells; T98G: NC, 83 ± 5 cells), whereas a strongly inhibited invasive ability was observed in *HDAC1* knockdown cells (U251: 42 ± 3 cells; T98G: 40 ± 6 cells; Figure 4C and 4D, respectively).

To investigate the function of HDAC1 on cell adhesion to matrix, cell adhesion assay was performed in fibronectin-coated plates. Compared with the NC cells (69 ± 7 cells and 54 ± 2 cells), both HDAC1-shRNA-U251 (Figure 4E) and HDAC1-shRNA-T98G GBM cells (Figure 4F) showed significantly decreased adhesive ability (42 ± 3 cells and 22 ± 2 cells, respectively). Our results suggest a role of HDAC1 in the promotion of glioblastoma invasion.

### Identification of HDAC1-associated biological pathways by GSEA

**Figure 5:**
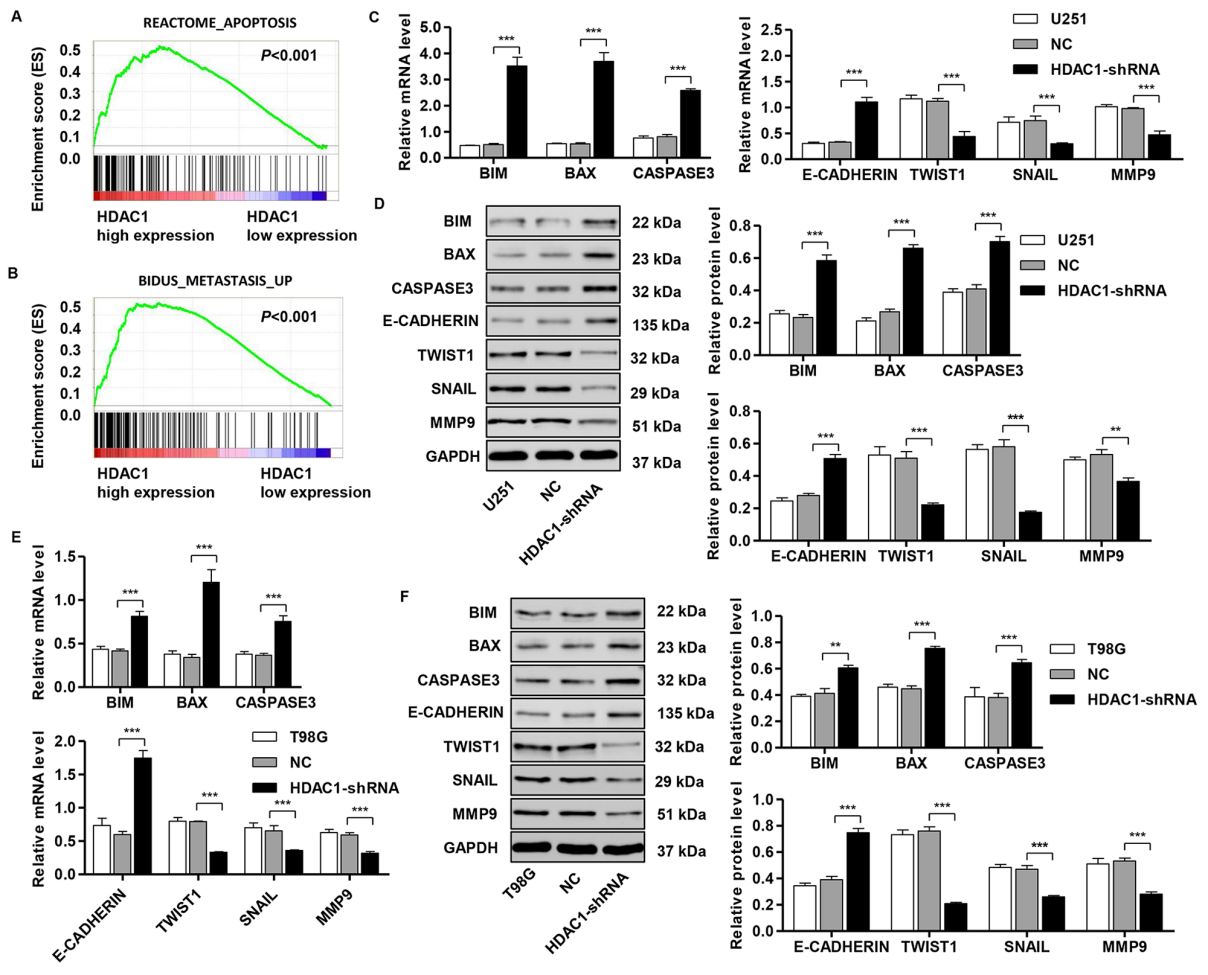
Mechanisms of HDAC1 exert their functions in glioma cell lines Ggene set enrichment analysis (GSEA) compared the HDAC1 higher expression group (red) with the HDAC1 lower expression group (blue) of glioma patients in TCGA dataset. Enrichment plots are shown for a set of activated gene pathway related to **(A)** apoptosis pathways and **(B)** metastasis pathways. Real-time PCR and Western blot analysis identified significant increases in BIM, BAX, cleaved CASPASE3 and E-CADHERIN, while decreases in TWIST1, SNAIL and MMP9 expression were found in **(C,D)** U251 and **(E,F)** T98G cells infected with pLVTHM-HDAC1-shRNA. Representative Western blots (left panel) and quantitative results (right panel) are shown. NC: pLVTHM-shRNA negative control infected cells. **P < 0.01, ***P <0.001 by the unpaired, two-tailed Student's t-test.

To assess the HDAC1-related pathways on an unbiased basis, we performed GSEA using data from the TCGA GBM cohort. The expression level of *HDAC1* gene was used as the phenotype label. As shown in Figure 5A and 5B, REACTOME_APOPTOSIS and BIDUS_METASTASIS_UP pathways were significantly associated with *HDAC1* expression in the TCGA GBM cohort (*P*<0.001).

To validate the GSEA analysis of HDAC1, we analyzed the mRNA and protein levels of the apoptosis and metastasis pathway-related factors in HDAC1-shRNA -infected U251 and T98G cells. As shown in Figure 5C and 5D, the mRNA and protein levels of apoptosis-related factors (BIM, BAX and cleaved CASPASE3) and an invasion-related factor (E-CADHERIN) were significantly higher in HDAC1-shRNA-infected U251 cells than in corresponding control cells (NC), while the invasion-related factors (TWIST1, SNAIL and MMP9) were remarkably lower in HDAC1-shRNA-infected U251 cells. Consistent with the functional characterization measured in U251 cells, T98G cells displayed an increase in apoptosis-related factors, but a decrease in invasion-related factors (Figure 5E and 5F).

### Knockdown of HDAC1 suppresses growth of tumor xenograft in nude mouse

**Figure 6:**
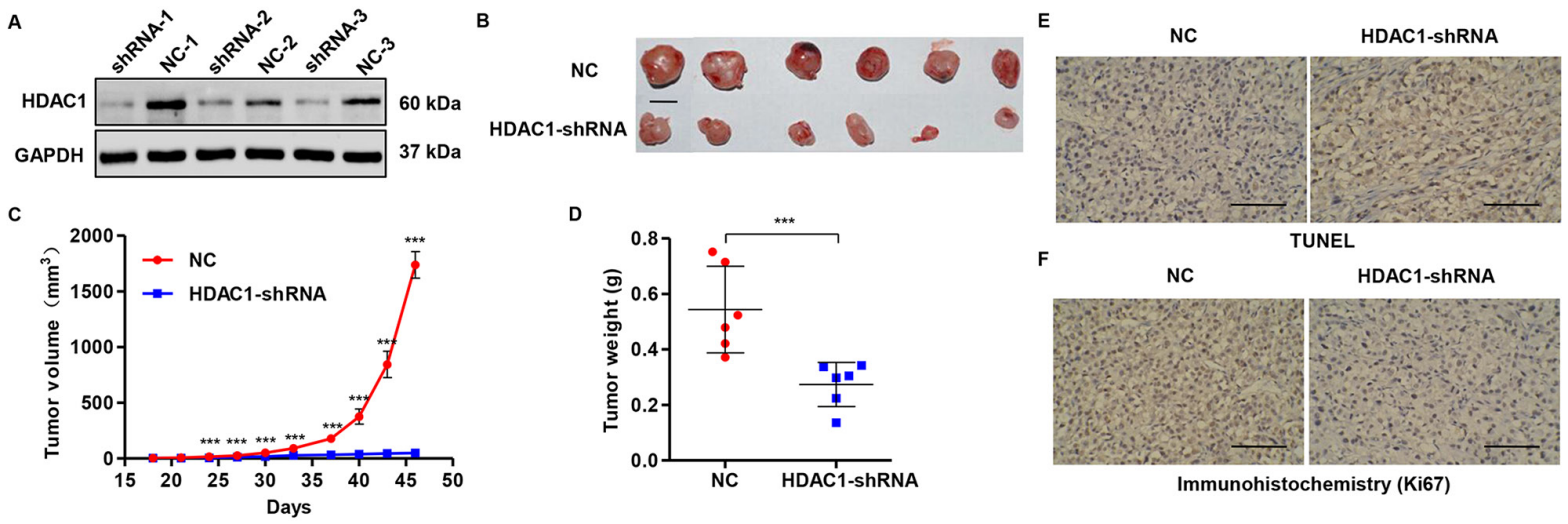
Knockdown of HDAC1 in glioma cells reduces tumor growth *in vivo* T98G cells infected with pLVTHM-shRNA negative control (NC) or pLVTHM-HDAC1-shRNA were subcutaneously injected in the armpits of nude mice. **(A)** HDAC1 expression was measured by Western blot. **(B)** *HDAC1* knockdown inhibits tumor growth in nude mouse xenograft model *in vivo*. Scale bar: 10 mm. **(C)** Tumor volume and **(D)** weight were also measured after *HDAC1* knockdown. Apoptosis and Ki67 expression were detected by **(E)** TUNEL and **(F)** immunohistochemistry assays. NC: pLVTHM-shRNA negative control infected cells. Scale bars: 100 μm. ***P < 0.001 by the unpaired, two-tailed Student's t-test.

To confirm whether *HDAC1* down-regulation in glioblastoma cells could inhibit the growth of tumor xenograft *in vivo*, stable T98G cells infected with pLVTHM-shRNA (negative control, NC) or pLVTHM-HDAC1-shRNA were subcutaneously injected in nude mice. HDAC1 levels in tumors formed from pLVTHM-HDAC1-shRNA infected T98G cells, exhibited 68.7% decreased expression of HDAC1 than in tumors from control cells infected with pLVTHM-shRNA (negative control, NC) (Figure 6A). Moreover, the tumors formed in pLVTHM-HDAC1-shRNA group were substantially smaller than those in the pLVTHM-shRNA (negative control, NC) group (Figure 6B and 6C). Mice in the HDAC1-shRNA group and the NC group were killed 46 days after inoculation, with the mean tumor weight was markedly lower in the pLVTHM-HDAC1-shRNA group (0.27 ± 0.08 g) and compared to the pLVTHM-shRNA (negative control, NC) group (0.54 ± 0.16 g), respectively (Figure 6D). Compared with the NC group, a notable increase of apoptotic cells and a significant decrease of Ki67-positive cells were observed in tumors formed from knockdown *HDAC1* cells by TUNEL (Figure 6E) and immunohistochemistry assay (Figure 6F). These results indicate that *HDAC1* knockdown inhibits growth of tumor xenograft in nude mouse.

## DISCUSSION

The expression of HDAC1 in different cancers has been reported recently [11–14, 26]. In the previous studies, people demonstrated that the patients with advanced stage, uncontrolled tumor cellular proliferation and poor prognosis showing an increased HDAC1 expression [27, 28], indicating that HDAC1 may as a molecular biomarker play an important role in glioma patients. Our data first confirmed that *HDAC1* mRNA levels were lower in normal brain tissues in comparison with that in glioma tissues, which was supported by glioma patients’ data from TCGA as well as Xinhua Hospital Affiliated to Shanghai Jiao Tong University School of Medicine, in which the increased HDAC1 protein expression was also found in glioma tissues compared with normal brain tissues. Moreover, HDAC1 expression was associated with WHO grade, MIB (%) and patients’ survival time. These data present here strongly favored the notions that HDAC1 may act as an oncogene in glioma tumorigenesis.

Mechanistic basis for the pathological and clinical observations in this study was further validated by assessing the biological functions of HDAC1 on glioma cell proliferation, apoptosis, migration and invasion. HDAC inhibitor preserves the acetylation status of proteins and induces renal cancer cell cycle arrest and apoptosis [29]. Clinical trials have been shown that HDAC inhibitors are potent anti-cancer drugs and have recently shown a significant therapeutic effect on glioma [30]. Consistent with a previous study [31], our data showed that *HDAC1* knockdown not only inhibited cell proliferation, migration, adhesion, and invasion in glioblastoma cell lines but also induced apoptosis and inhibited tumor growth *in vivo*. In addition, HDAC1-shRNA decreased the effect of CXCL1/GROα on migration and invasion, suggesting that HDAC1 is also involved in CXCL1/GROα-mediated prostate cancer progression [32]. Thus, the increased expression of HDAC1 may be associated with the abnormal cellular proliferation and invasiveness potential of gliomas. Although knockdown of *HDAC1* inhibited the cell invasion of glioblastoma, it may, in part, due to the inhibition of cell proliferation of glioblastoma. Therefore, a comprehensive effect of *HDAC1* downregulation on glioblastoma cell invasion needs further investigation.

The exact pathway that HDAC1 may involve in gliomas remains unclear. Our GSEA results indicated that *HDAC1* overexpression was positively correlated with apoptosis and metastasis pathways. To validate the GSEA analysis of HDAC1, we analyzed the mRNA and protein levels of the apoptosis and invasion pathway-related factors in HDAC1-shRNA-infected glioblastoma cells. We observed that the expression of apoptosis-related factors (BIM, BAX and cleaved CASPASE3) and an invasion-related factor (E-CADHERIN) were higher, while the expression of related invasion factors (TWIST1, SNAIL and MMP9) were lower in glioblastoma cells with HDAC1-shRNA infection. These data suggest that these factors participate in HDAC1-induced glioma progression. Apoptosis is a well-orchestrated cellular mechanism regarding a process of programmed cell death that coordinates cell proliferation and cell death. A recent research presented evidence that HDAC1 downregulation results in the increased histone acetylation of Fas promoter, following the upregulation of Fas expression and consequently, increases sensitivity of T cells to activation-induced cell death [33]. Upon apoptosis, the “activator” Bim activates Bax and Bak to mediate cytochrome *c* efflux, leading to caspase activation [34]. HDAC inhibition suppressed p53-dependent activation of Bax, thus preventing post-mitochondrial events, including cleavage of Caspase-9 and Caspase-3 [35]. However, in contrast to our findings, downregulation of HDAC1 by RNAi suppressed TGF-β1-induced apoptosis in AML-12 and primary mouse hepatocytes, indicating that HDAC1 functions as a proapoptotic factor in TGF-β1-induced apoptosis [36]. These findings may actually provide us with necessary insights into the role of HDAC1 and its potential mechanisms in apoptosis. Cancer proliferation and invasion are the leading causes of mortality in patients with glioma. Previous studies have observed that HDAC inhibitors significantly suppressed the migration and invasion of prostate cancer cells, which is corrected by knockdown of E-cadherin, suggesting a possible mechanism of E-cadherin in HDAC inhibitors regulating migration and invasion [37]. In addition, silencing of E-cadherin by a transcriptional repressor complex containing Snail and HDAC1 in highly metastatic cells indicates the implication of HDAC with invasion progression and provides preclinical evidence that HDACs can act as a novel target for anti-invasion therapy [38]. Twist-related protein 1 (Twist1), a transcription factor of the basic helix-loop-helix class, is reported to promotes cancer invasion, induces epithelial-mesenchymal transition (EMT) [39], and reduces ER transcript levels through interacting with HDAC1 at the ER promoter, leading to histone deacetylation and chromatin condensation [40]. Matrix metalloprotein-9 (MMP-9) acts as an important oncogene that promotes cancer cell migration and migration and mediates extracellular matrix (ECM) degradation [41]. Prior research has also demonstrated that increased MMP-9 expression resulted in a poor prognosis in a variety of cancers [42, 43]. In agreement with our results *HDAC1* siRNA inhibited invasion by decreasing *MMP9* mRNA expression, while overexpression of *HDAC1* increased invasion and MMP9 expression in breast cancer [44]. In the present study, *HDAC1* knockdown significantly increased the expression of BIM, BAX, cleaved CASPASE3 and E-CADHERIN, and suppressed the expression of TWIST1, SNAIL and MMP9, which could be interpreted to be pro-apoptosis and anti-invasion effects of *HDAC1* shRNA in glioma cells. Further investigation is required to elucidate the detailed mechanisms by which HDAC1 increases or decreases these proteins. Therefore, HDAC1 may therefore be considered an oncogene and a poor indicator of development in patients with glioma, and may serve as a therapeutic target in the future.

## MATERIALS AND METHODS

### Patient samples

Tumor tissues were collected from 105 glioma patients (I: 9, II: 14, III: 23 and IV: 59), and 25 normal brain samples were collected from patients with temporal lobe epilepsy who were admitted to our hospital from January 2010 to December 2012. Tumor and normal brain tissues were immediately snap-frozen in liquid nitrogen and stored at -80°C until total RNA was extracted. The patients’ clinical characteristics such as age, gender, tumor size, WHO grade, and MIB (%), were collected for statistical analysis. Ethical approval for the study was provided by the independent ethics committee of Xinhua Hospital, affiliated with Shanghai Jiao Tong University School of Medicine. Informed and written consent were obtained from all patients or their advisors according to the ethics committee guidelines.

### Bioinformatics analysis

RNA-Seq data from 528 glioma and 10 normal brain tissues were downloaded from TCGA following approval of this project by the consortium. To validate the correlation of HDAC1 and Pathways in cancer, especially apoptosis and metastasis pathways involved in glioma pathogenesis, a GBM cohort downloaded from TCGA was analyzed by GSEA as previously described. Gene set enrichment analysis was performed using the GSEA software, Version 2.0.1, obtained from the Broad Institute (http://www.broad.mit.edu/gsea). The nominal *P* value and normalized enrichment score (NES) were used to sort the pathways enriched in each phenotype.

### Cell culture

SHG44, U87, U251, U373 and T98G glioblastoma cell lines were obtained from the cell bank of the Shanghai Biology Institute, Chinese Academy of Science. All cells were cultured in Eagle's Minimum Essential Medium (MEM; Hyclone, Logan, UT, USA), with the exception of SHG44 and U251 cells, which were cultured in Dulbecco's modified Eagle's medium (DMEM; Hyclone). All culture media were supplemented with 10% fetal bovine serum (FBS, Hyclone) and 1% antibiotic (penicillin/streptomycin, Gibco). All cells were maintained at 37°C in 5% CO_2_.

### Vector construction and virus infection

Oligonucleotides encoding shRNA directed against human *HDAC1* (point 355-377 HDAC1-shRNA, 5’-GGACTGTCCAGTATTCGAT-3’) and scramble shRNA were purchased from SangonBiotech, Shanghai. The shRNA sequences were cloned into the pLVTHM using *Mlu*I and *Cla*I. The scramble shRNA was cloned into the pLVTHM vector and used as negative control (NC). The constructs were then co-transfected into HEK 293T cells with lentiviral transfection system by using lipofectamine 2000 (Invitrogen Life Technologies, Gaithersburg, MD, USA) according to the manufacturer's instruction. After 48 h transfection, viruses were collected and used to infect U251 and T98G cells for 6 hours.

### Cell proliferation assay

Cell proliferation was performed using Cell Counting Kit-8 (CCK-8, Dojindo, Kyushu, Japan) assay, as previously described [45]. U251 and T98G cells were seeded in 96-well plates at the density of 3×10^3^ cells per well and cultured at 37°C in 5% CO_2_ for 12 hours. After infection for 0, 24, 48 and 72 h, CCK-8 solution was added to each well and incubated for another 1 hour. Cell proliferation was determined by scanning with a microplate reader (Bio-Rad Laboratories, Hercules, CA, USA) at 450 nm. Each experiment was performed in triplicate.

### Cell apoptosis assay

Apoptosis was determined by flow cytometer (BD biosciences, San Diego, CA, USA), and an annexin-V fluorescein isothiocyanate (FITC)/Propidium Iodide (PI) double-stain assay was performed in accordance with the manufacturer's protocol (BioVision, Mountain View, CA, USA). After infection for 48 hours, U251 and T98G cells were washed three times with phosphate buffer saline (PBS), trypsinized, centrifuged (400 x g at room temperature) for 5 min, adjusted to 5×10^4^/ml and suspended in binding buffer containing Annexin V-FITC and PI. After incubation for 20 min at room temperature in the dark, the fluorescent intensity was measured using a flow cytometer (BD Biosciences, San Jose, CA, USA). Each experiment was performed in triplicate.

### *In vitro* migration and invasion assays

Invasion assays were performed using a Transwell chamber (Greiner Bio-One, Frickenhausen, Germany) coated with Matrigel (BD Biosciences, San Jose, CA, USA) as described in the manufacturer's protocol. After infection for 48 hours, 1×10^5^ cells in 500 μL serum-free MEM or DMEM were seeded into the upper well of the chamber. The lower chamber was filled with 750 μL MEM or DMEM containing 10% FBS. After the cells were incubated for 48 hours at 37°C in a 5% CO_2_ atmosphere, cells adhering to the lower surface were fixed with 4% paraformaldehyde and stained with crystal violet solution for 30 min. Cells on the top surface of the insert were removed with a cotton swab and counted under a microscope (Olympus Corporation, Tokyo, Japan) in five fields (× 200). The procedure for the cell migration assay was similar to the cell invasion assay, except that the transwell membranes were not pre-coated with Matrigel (BD Biosciences, San Jose, CA, USA). Each experiment was performed in triplicate.

### *In vitro* cell adhesion assay

After infection for 48 hours, U251 and T98G cells were seeded on fibronectin-coated 12-plate microplate at a density of 1×10^5^ cells per well and then incubated for 1 hour. The supernatant was discarded, and cells were washed three times using PBS. 4% paraformaldehyde was supplemented for 15 minutes, and cells were Giemsa stained for 30 minutes. The adherent cells were photographed and counted under a microscope (Olympus Corporation, Tokyo, Japan) in five fields (× 200). Each experiment was performed in triplicate.

### RNA extraction and RT-PCR

Total RNA was extracted from glioma, normal brain tissues, and five glioblastoma cells using Trizol reagent (Thermo Fisher Scientific, Rockford, IL, USA) according to the manufacturer's instructions. Complementary DNA was synthesized with cDNA synthesis kit (Thermo Fisher Scientific, Rockford, IL, USA). Real-time quantitative PCR was performed using a standard SYBR Green PCR kit (Thermo Fisher Scientific, Rockford, IL, USA) on an ABI 7500 Real-Time PCR machine (Applied Biosystems, Foster City, CA, USA). Primer sequences were listed in Table 2. GAPDH was used as control of the input RNA level. The gene expression was calculated using the ΔΔ Ct method. Each experiment was performed in triplicate.

**Table 2:** Primes sequences used in this study

Gene	Sequences
*HDAC1*-forward	5′-GCTCCACATCAGTCCTTCC-3′
*HDAC1*-reverse	5′-GGTCGTCTTCGTCCTCATC-3′
*BAX*-forward	5′-AGCTGAGCGAGTGTCTCAAG-3′
*BAX*-reverse	5′-TGTCCAGCCCATGATGGTTC-3′
*CASPASE3*-forward	5′-AACTGGACTGTGGCATTGAG-3′
*CASPASE3*-reverse	5′-ACAAAGCGACTGGATGAACC-3′
*BIM*-forward	5′-CCACCAGCACCATAGAAG-3′
*BIM*-reverse	5′-GAGCAGGCACAGAGAAAG-3′
*TWIST1*-forward	5′-AGTCCGCAGTCTTACGAG-3′
*TWIST1*-reverse	5′-GCTTGCCATCTTGGAGTC-3′
*SNAIL*-forward	5′-TTCCTGAGCTGGCCTGTCTG-3′
*SNAIL*-reverse	5′-TGGCCTGAGGGTTCCTTGTG-3′
*E-CADHERIN*-forward	5′-GAGAACGCATTGCCACATACAC-3′
*E-CADHERIN*-reverse	5′-AAGAGCACCTTCCATGACAGAC-3′
*MMP9*-forward	5′-AAGGGCGTCGTGGTTCCAACTC-3′
*MMP9*-reverse	5′-AGCATTGCCGTCCTGGGTGTAG-3′
*GAPDH*-forward	5′-CACCCACTCCTCCACCTTTG-3′
*GAPDH*-reverse	5′-CCACCACCCTGTTGCTGTAG-3′

### Western blotting analysis

Glioma tissues and cells were harvested, washed twice with PBS, lysed in ice-cold radioimmunoprecipitation assay buffer (RIPA, JRDUN Biotechnology, Shanghai, China) with a freshly added 0.01% protease inhibitor cocktail (Sigma-Aldrich, St. Louis, MO, USA) and incubated on ice for 30 minutes. Protein concentration was measured by bicinchoninic acid (BCA) protein assay kit (Pierce, Rockford, IL, USA). A total of 30 μg of protein was subjected to electrophoresis using SDS-PAGE and transferred electrophoretically to a nitrocellulose membrane (Millipore, Bedford, USA). Blots were visualized using enhanced chemiluminescence (ECL, Millipore, Billerica, MA, USA) after antibody binding. Antibodies against HDAC1, BIM, BAX, cleaved CASPASE3, MMP9 and TWIST1 were purchased from Abcam (Cambridge, MA, USA); antibodies against SNAIL, E-CADHERIN and GAPDH were purchased from Cell Signaling Technology Biotech (Danvers, MA, USA). The bands were quantified by the densitometry with Image J software (NIH, USA).

### Growth of cells in athymic nude mice and tumor size determination

T98G cells infected with pLVTHM-shRNA negative control (NC) or pLVTHM-HDAC1-shRNA were trypsinized, washed and re-suspended in DMEM without FBS. 12 male athymic nude mice (SLAC Laboratory Animal Center, Shanghai, China) were randomly divided into 2 groups (6 mice/group), and 2×10^6^ T98G cells were subcutaneously injected into the right armpit of the mice. At 46 days after injection, the mice were euthanized and the tumors were excised and weighed. The excised tumor tissues were formalin-fixed, paraffin-embedded, sectioned and then analyzed with TUNEL assay (Roche Applied Science, Mannheim, Germany) or Ki67 immunostaining (Abcam, Cambridge, MA, USA).

### Immunohistochemistry

Tissue sections were initially treated for deparaffinization and hydration and then heated in EDTA (pH 8.0) and antigen-retrieved in 10 mm citrate buffer for 5 minutes at 100°C. The reaction of HDAC1 or Ki67 antibody (Abcam, Cambridge, MA, USA) took place for 1 hour at room temperature, following incubation with biotin-labeled secondary antibodies. Slides were then developed using 3,3-diaminobenzidine (DAB; Shanghai Long Island, Co., LTD, China) solution and counterstained with hematoxylinstaining (BASO, China). Immunohistochemical signals were calculated with positive staining cells under a microscope (Olympus Corporation, Tokyo, Japan) with magnification of ×200.

### Statistical analyses

The results are presented as the mean value ± S.D. GraphPad Prism (version 5.0, GraphPad Software, La Jolla, CA, USA) software was used for statistical analyses. Statistical significance was determined by the unpaired, two-tailed Student's t-test and one-way ANOVA analysis. Differences were considered statistically significant if *P* < 0.05.

## Abbreviations

HDACs: histone deacetylases
WHO: World Health Organization
GBM: glioblastoma multiform
GSEA: gene set enrichment analysis
TCGA: The Cancer Genome Atlas
DNMT: DNA methyl transferases
PBS: phosphate buffer saline.
